# Canada

**Published:** 1896-07

**Authors:** 


*Canada.* Veterinarian W. B. Campbell, of Berlin, Ontario, reports an outbreak of contagious garget in a herd of cattle in his district. Two of these animals were affected within twelve hours of each other. They were in bank barns, poorly ventilated, no separation of the animals, many of the roots fed stored in the same place. Directly in front of the cows twenty hogs were kept. A large percentage of the herd of twelve were affected. Under saline purges, fomentations, camphor liniment, and the use of the siphon, they yielded tardily to treatment.

